# Correction to: ‘Infrastructure construction without information exchange: the trail clearing mechanism in *Atta* leafcutter ants’

**DOI:** 10.1098/rspb.2019.0265

**Published:** 2019-02-13

**Authors:** Thomas Bochynek, Martin Burd, Christoph Kleineidam, Bernd Meyer

*Proc. R. Soc. B* **286**, 20182539. (Published Online 23 January 2019) (doi:10.1098/rspb.2018.2539)

**Corrected funding statement**

T.B. was supported by two Monash University postgraduate scholarships and by CSIRO Data61. Model development and fieldwork were partially supported by Australian Research Council grants no. DP140103946 to M.B. and no. DP110101413 to B.M. Laboratory work was supported by Universities Australia-DAAD project 57219808 ‘Division of Labour and Collective Homeostasis in Dynamic Environments’ and by DFG Centre of Excellence 2117 ‘Centre for the Advanced Study of Collective Behaviour’ (ID: 422037984).

